# Multifunctionalizing the marine diatom *Phaeodactylum tricornutum* for sustainable co-production of omega-3 long chain polyunsaturated fatty acids and recombinant phytase

**DOI:** 10.1038/s41598-019-47875-1

**Published:** 2019-08-07

**Authors:** Alex Pudney, Chiara Gandini, Chloe K. Economou, Richard Smith, Paul Goddard, Johnathan A. Napier, Andrew Spicer, Olga Sayanova

**Affiliations:** 1 Algenuity, Eden Laboratory, Broadmead Road, Stewartby, BEDS MK43 9ND UK; 2 Department of Plant Sciences, Rothamsted Research, Harpenden, Herts AL5 2JQ UK; 3 School of Biological and Chemical Sciences, Queen Mary University of London, Mile End Road, London, E1 4NS UK; 4 Amalga Technologies Ltd, 80 Park Road, Hampton Wick, Kingston on Thames, Surrey, KT14AY UK

**Keywords:** Biotechnology, Metabolic engineering

## Abstract

There is an urgent requirement for sustainable sources of food and feed due to world population growth. Aquaculture relies heavily on the fish meal and fish oils derived from capture fisheries, challenging sustainability of the production system. Furthermore, substitution of fish oil with vegetable oil and fish meal with plant seed meals in aquaculture feeds reduces the levels of valuable omega-3 long chain polyunsaturated fatty acids such as eicosapentaenoic (EPA) and docosahexaenoic (DHA) acids, and lowers the nutritional value due to the presence of phytate. Addition of exogenous phytase to fish feed is beneficial for enhancing animal health and reducing phosphorus pollution. We have engineered the marine diatom *Phaeodactylum tricornutum*, accumulating high levels of EPA and DHA together with recombinant proteins: the fungal *Aspergillus niger* PhyA or the bacterial *Escherichia coli* AppA phytases. The removal of the N-terminal signal peptide further increased phytase activity. Strains engineered with fcpA and CIP1 promoters showed the highest level of phytase activity. The best engineered strain achieved up to 40,000 phytase activity units (FTU) per gram of soluble protein, thus demonstrating the feasibility of development of multifunctionalized microalgae to simultaneously produce industrially useful proteins and fatty acids to meet the demand of intensive fish farming activity.

## Introduction

The rise in world population is driving demand for high-quality protein and hydrocarbon production for both human and animal consumption. Protein productivities from conventional sources such as terrestrial animals, crops and aquaculture are reaching their limits of sustainability. Aquaculture is one of the fastest growing sectors of the food industry, driven by ever-increasing consumer demand and now supplies the majority of fish for human consumption: in 2014, 74 million tons of fish consumed by humans was produced by fish-farming^1^. Farmed fish, however, require feeds containing fish meal and fish oil as they supply omega-3 long chain polyunsaturated fatty acids (LC-PUFAs), such as eicosapentaenoic (EPA) and docosahexaenoic (DHA) acids, both of which are essential for optimal nutrition and stress tolerance of marine fish, especially at the larval and juvenile stages^2^. Aquaculture’s consumption of fish meal and fish oil has more than doubled over the past decade^3^. It is well known that the industry’s use of fish meal has increasingly become economically and environmentally unsustainable^4^. Recently, the aquaculture industry has started to replace fish oil and fish meal with plant seed meals and vegetable oils^5^. However, one of the major constraints on using plant proteins in animal feed, is the presence of anti-nutritional factors such as phytate. Phytic acid (myo-inositol hexakisphosphate or IP_6_) is the primary storage form of phosphate in plants, accounting for ~80% of the total phosphorus^6^. Monogastric animals such as poultry, swine and fish are incapable of digesting phytate from plant-derived feeds as they lack a phytase enzyme^7^. In addition, phytate can chelate with minerals such as calcium, magnesium, zinc, copper and iron and essential amino acids to form insoluble salts^8^. This results in decreased phosphorus bioavailability and has a negative effect on growth rates and mineral uptake. Therefore, dietary supplementation with bioavailable phosphate is required to achieve optimal animal growth performance. Furthermore, the poor digestibility of phosphorous from phytate in animal feeds creates animal waste comparatively rich in phytate which can be released by bacterial or fungal action to become an environmental pollutant.

For these reasons, phytase enzyme is now widely used as an animal feed additive in diets of swine, poultry and fish to increase the uptake of phytate-derived phosphorus and reduce the environmental burden resulting from phytate rich waste. Phytases (*myo*-inositol hexakisphosphate phosphohydrolases) belong to a group of phosphatases (EC 3.1.3.8), catalysing the sequential dephosphorylation of phytate to *myo*-inositol and inorganic phosphate^9^. The fungal *Aspergillus niger* PhyA and the bacterial *Escherichia coli* AppA are both used as supplements in fish feed to improve availability of P_i_ and trace elements, maximize digestibility and growth, and also to reduce phosphate pollution into the aquatic environment^10^. However, the use of exogenous phytases is associated with high production costs and there is an urgent requirement for sustainable production systems. Recently, expression of PhyA and AppA in transgenic plants demonstrated the feasibility of an alternative approach to the production of phytase for commercial use^11–15^. In addition, the green microalga, *Chlamydomonas reinhardtii*, has been genetically engineered to accumulate recombinant PhyA^16^ and AppA^17^ for use as food additives without the need for protein purification.

The direct use of microalgae in aquaculture feeds is well established and confers numerous benefits including improved nutrition and digestibility^18,19^. Microalgae are a highly nutritious aquaculture feed owing to their favourable composition of micro- and macro-nutrients, notably including a high protein content and additional presence of EPA and DHA. *Phaeodactylum tricornutum* is a marine diatom that accumulates up to 35% EPA and traces of DHA. It contains 49% protein and is used to feed fish larvae and molluscs. It is rapidly cultivable, is genetically tractable, has a fully-sequenced genome^20^ and a rapidly expanding molecular toolbox making it an appealing system for metabolic engineering. In addition, recombinant protein production has been reported^21,22^ demonstrating clear opportunities for multi-functionalization where expression of a recombinant protein, or production of a valuable metabolite adds value to an already nutritionally and functionally valuable biomass.

Recently we engineered *P*. *tricornutum* to accumulate enhanced levels of DHA by over-expressing the Δ5-elongase from the picoalga *Ostreococcus tauri*^23^ and demonstrated the potential of this transgenic strain for industrial production of omega-3 LC-PUFAs^24^. DHA content in the transgenic Pt_OtElo5 strain was increased up to eight-fold compared to that of wild type (WT) cells.

In this study, we have implemented our previously established microalgal system^23^ to produce transformed *P*. *tricornutum* strains expressing the two most widely used phytases in the fish food industry, from *A*. *niger* and *E*. *coli*, respectively. The activity of different promoters was investigated to achieve the best expression levels. We demonstrated for the first time the feasibility of producing transgenic microalgal strains accumulating both high value omega-3 LC-PUFA EPA and DHA and recombinant proteins, such as the enzyme phytase to tackle the problems regarding phosphorus deficiency and general animal health.

## Results

### Generation of transgenic *P*. *tricornutum* strains co-expressing phytase genes and Δ5-elongase from the picoalga *O. tauri*

To test the expression of transgenes in *P*. *tricornutum*, coding sequences for *O*. *tauri* Δ5-elongase, OtElo5^25^, and a phytase from either *E*. *coli* (AppA)^26^ or *A*. *niger* (PhyA)^27^ were cloned into the modified pPhOS2 vector^23^. We have previously described the successful expression of OtElo5 using the highly-expressed fcpA promoter of the endogenous fcpA gene. This gene encodes a member of the light harvesting complex superfamily, the fucoxanthin chlorophyll a-binding protein^28^. However, fcp promoters may not be suitable for the expression of heterologous transgenes in all processes, owing to the presence of light-responsive cis-regulatory elements. As an alternative, Seo *et al*., 2015 reported that use of the endogenous elongation factor 2 (EF2) promoter isolated from *P*. *tricornutum* resulted in a 1.2-fold increase in transgene expression compared with the fcp promoter in light conditions, and that EF2-mediated expression was stable in *P*. *tricornutum* throughout light and dark cycles^29^. In addition, a study characterising transgene expression using an algal virus promoter of a putative replication-associated (VP3) gene from *Chaetoceros lorenzianus*-infecting DNA virus (ClP1) showed that promoter activity remained stable through a photoperiod, and in cells grown in low nutrient culture conditions^30^. Further, the ClP1 promoter activity levels were higher than endogenous fcp and EF2 promoters, suggesting that CIP1 promoter may be valuable for biotechnological applications.

**Figure 1:**
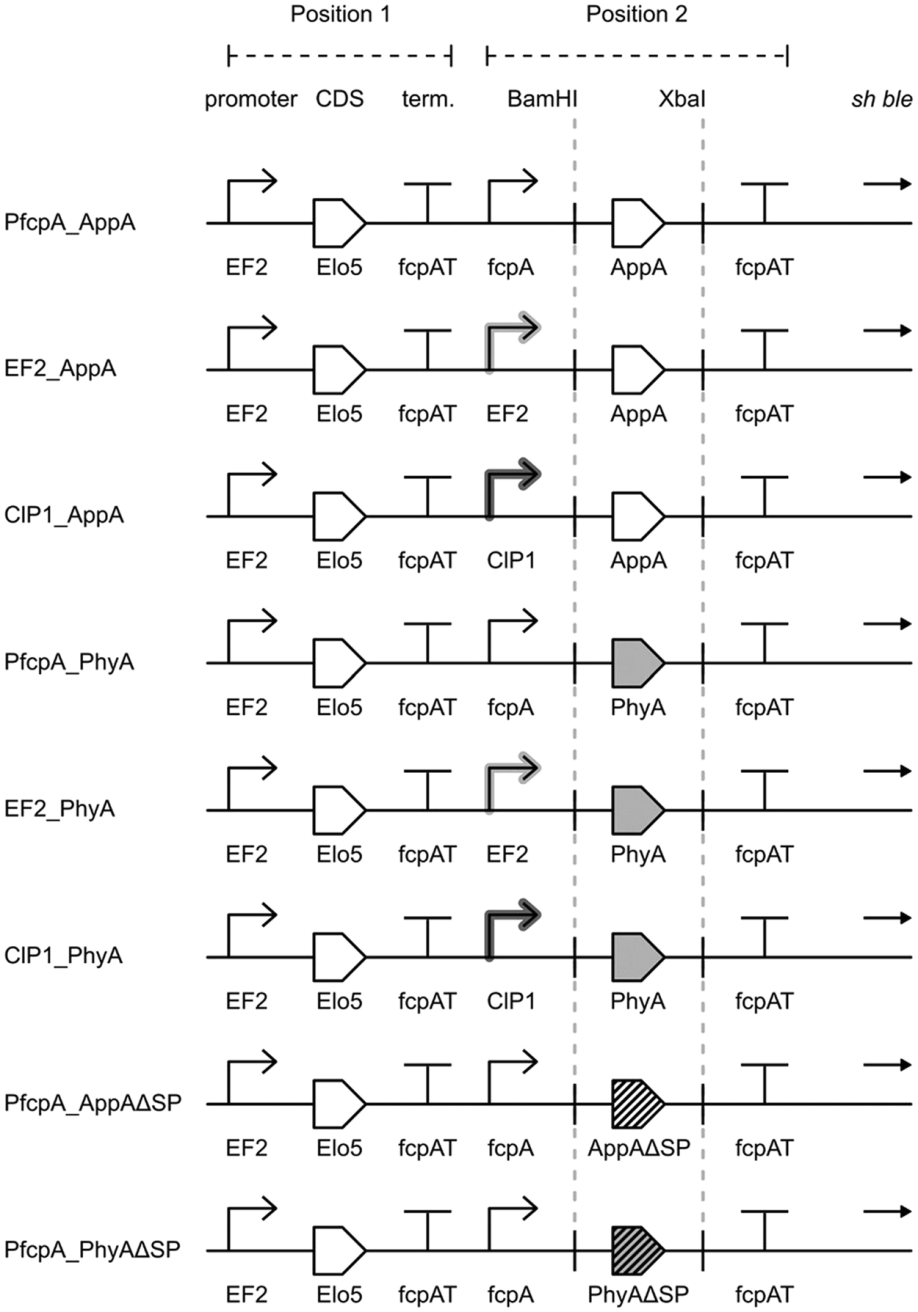
Schematic representation of the constructs used to transform *P*. *tricornutum*. The OtElo5 gene was cloned into position 1 under control of the native EF2 promoter^29^. Codon-optimised *A*. *niger* phytase (PhyA) or *E*. *coli* phytase (AppA) were cloned as *BamHI- XbaI* fragments into position 2 under control of either endogenous fcpA^31^ or EF2 promoters, or the ClP1 viral promoter^30^. Abbreviations: fcpAT, native *P*. *tricornutum* fcpA terminator; AppAΔSP, AppA without the putative N-terminal signal peptide; PhyAΔSP, PhyA without the putative N-terminal signal peptide.

To identify more-optimal promoters for heterologous phytase expression in *P*. *tricornutum*, we firstly designed a two-gene expression vector, pPt_EF2_Elo5 (Supplementary Fig. S1). Codon-optimized coding sequences for either *AppA* or *PhyA* full-length phytase genes were subsequently inserted into the pPt_EF2_Elo5 vector, under the control of either native fcpA^31^ and EF2^29^ promoters, or the ClP1 viral promoter^30^ (Fig. 1). These plasmids were introduced into *P*. *tricornutum* via biolistics and multiple independent zeocin-resistant transgenic lines were obtained. Individual clones were screened by PCR for the presence of both OtElo5 and phytase transgenes and positive lines were used to inoculate cultures for further testing for both enhanced DHA production and phytase expression.

### Determination of phytase activity in transgenic *P*. *tricornutum*

Biomass harvested from late log-phase cultures of PCR-positive *P*. *tricornutum* strains was lysed by sonication and complete lysis was confirmed by microscopy (Supplementary Fig. S2). The soluble fraction was tested for the presence of phytase by enzyme assay, specifically developed for use in diatoms.

Phytase activity in commercial feed products is expressed in standard phytase activity units (FTU), where one unit of phytase corresponds to the amount of enzyme that releases 1 µmol of inorganic phosphate (P_i_) from inositol hexakisphosphate (IP_6_) per minute at 37 °C. Accurate determination of phytase activity in a cell extract requires the ability to differentiate the phosphate released by phytase from the contaminating activity of endogenous phosphatases and the promiscuous degradation of IP_6_-competing substrates^32^. The use of a colorimetric method, which determines only the total Pi released, is therefore not an optimal choice for this application. Instead, the phytase assay we adopted is based on a previously reported method^33^ in which the enzyme activity is determined directly, by measuring the reduction in optical density of an IP_6_: lysozyme turbid substrate complex (OD_600_ nm/min/mg). We used an iterative trial and error approach to identify an optimised reaction mix which reproducibly gave a stable, turbid substrate complex (OD_600_ nm > 3.0), that also exhibited a minimal decrease in OD_600_ nm (approximately 6%) after 60 minutes’ incubation at 37 °C in the presence of *P*. *tricornutum* cell extracts. This was achieved by testing reaction components in the range of 0.2 to 0.4 mM phytic acid and 1.2 to 2.2 mg/mL lysozyme. The improved phytase reaction mix comprised a ratio of 0.4 mM IP_6_ to 1.7 mg/mL lysozyme in a 50 mM glycine solution at pH 2.5.

**Figure 2:**
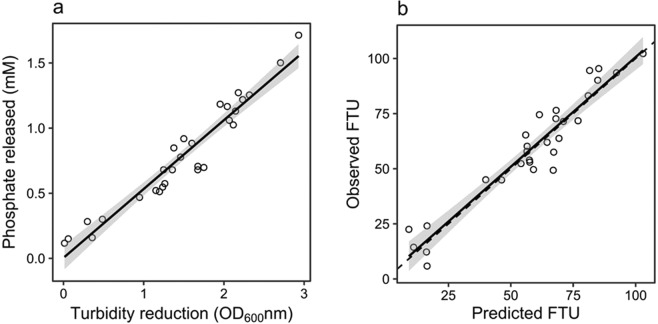
Regression model for enumeration of phytase activity (FTU). (**a**) A prediction model of the linear relationship between the amount of phosphate released and the reduction in turbidity of the substrate complex was used to calculate phytase activity in transgenic *P*. *tricornutum*. (**b**) The model accuracy was confirmed with the same data used in its construction by plotting observed FTU (colorimetrically-determined) against predicted FTU (calculated from the linear portion of each assay curve) and comparing with the 1:1 line (dashed line). Shaded regions are 95% confidence intervals of their respective regression lines (solid lines).

To convert phytase activity from OD_600_ nm/min/mg to FTU, it was necessary to correlate the reduction in OD_600_ nm with the corresponding amount of phosphate released using a regression model (Fig. 2a). Data for the prediction model used to calculate FTU was collected by using dilutions of a commercially supplied phytase to achieve different rates of substrate hydrolysis. Reactions were stopped at various time points during the linear phase, and total phosphate content was quantified using the ascorbic acid method^32^. Model accuracy was confirmed by checking predictions using the original assay data. The resulting plot of observed FTU against predicted FTU^34^ (Fig. 2b), showed no model bias for phytase activity predictions in the range of 9.16 to 102.97 FTU/mg soluble protein (a = 1.186, p = 0.756; b = 0.994, p = 0.926). The associated residual standard error of the model (7.81 FTU) is consistent with observed variability within the assay itself. Although wild-type *P*. *tricornutum* appeared to exhibit an endogenous phytase activity using this assay, the level was not greater than the standard error of the prediction model, and so is likely just an artefact of the assay method. Therefore, a robust protocol was developed to assay phytase activity in cell extracts of *P*. *tricornutum*.

### Promoter testing to optimise phytase expression

**Figure 3:**
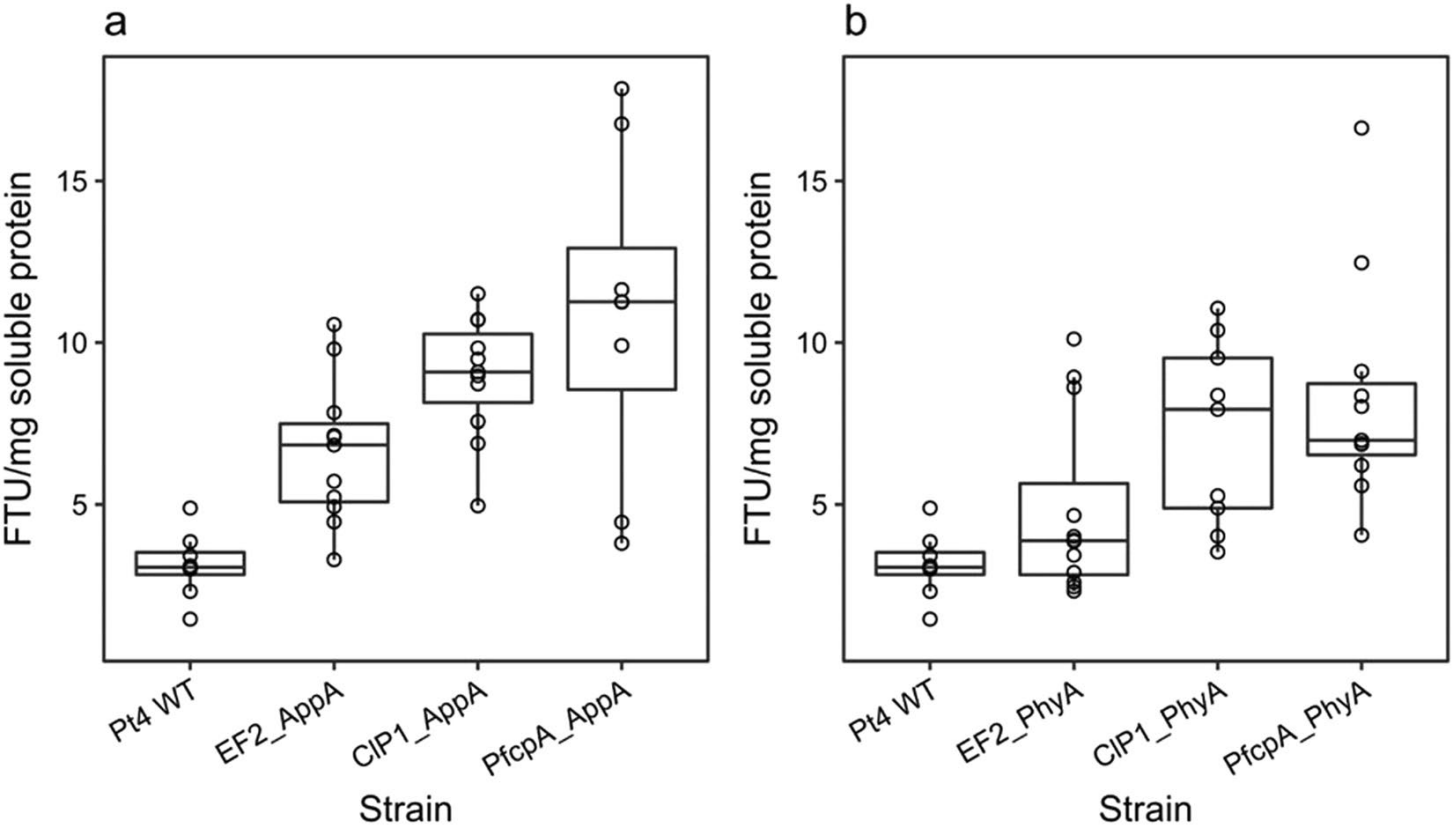
Comparison of phytase activities from independent transgenic strains of *P*. *tricornutum* using different promoters. (**a**) Clones expressing *E*. *coli* AppA. (**b**) Clones expressing *A*. *niger* PhyA. Data shown as boxplots with the line at the median. Values are averages of measurements of at least three independent cultures, ±standard error.

To examine whether intracellular accumulation of transgenic phytase could be improved by altering promoter choice, the expression of AppA or PhyA was tested with either *P*. *tricornutum* native promoters fcpA and EF2, or with the ClP1 viral promoter (Fig. 3).

Comparison of phytase activities from *P*. *tricornutum* transformants expressing either AppA or PhyA showed that use of different promoters influenced the level of phytase activity observed between strains. For both phytases, the difference in average mean enzyme activity between the fcpA and EF2 promoter groups was statistically significant (p < 0.05, LSD), consistent with the observation that better phytase expressors were isolated from the fcpA promoter pool, compared with EF2. The difference between fcpA and ClP1 group mean phytase activities was not statistically significant for either AppA or PhyA groups (p > 0.05, LSD). Therefore, either of these promoters can be recommended as preferential choices for transgene expression in *P*. *tricornutum* when compared with EF2.

For both sets of transgenic phytase strains, the difference in enzyme activities within promoter groups was greater than the difference in the median observation between groups. For example, there was a four-fold difference in the range of assayable PhyA activities for strains isolated from the fcpA promoter pool, which included not only the two best-expressing strains for *A*. *niger* phytase (16.64 and 12.47 FTU/mg soluble protein, respectively), but also strains whose assayable phytase activity was not distinguishable from wild-type *P*. *tricornutum* (Fig. 3b). This variation in the expression of transgenes from the nuclear chromosome is likely due to positional effect of the transgene integration site or epigenetic-derived transgene silencing^35,36^.

### Deletion of N-terminal signal peptide improves phytase activity in transgenic *P*. *tricornutum*

Further optimisation of phytase activity was achieved by removing the respective DNA sequences corresponding to putative N-terminal secretion signal peptides (SP) in both AppA and PhyA, generating AppAΔSP and PhyAΔSP constructs respectively (Fig. 1). Following transformation, several PCR-positive transformants were further evaluated by phytase assay to assess any improvement in enzyme activity.

**Figure 4:**
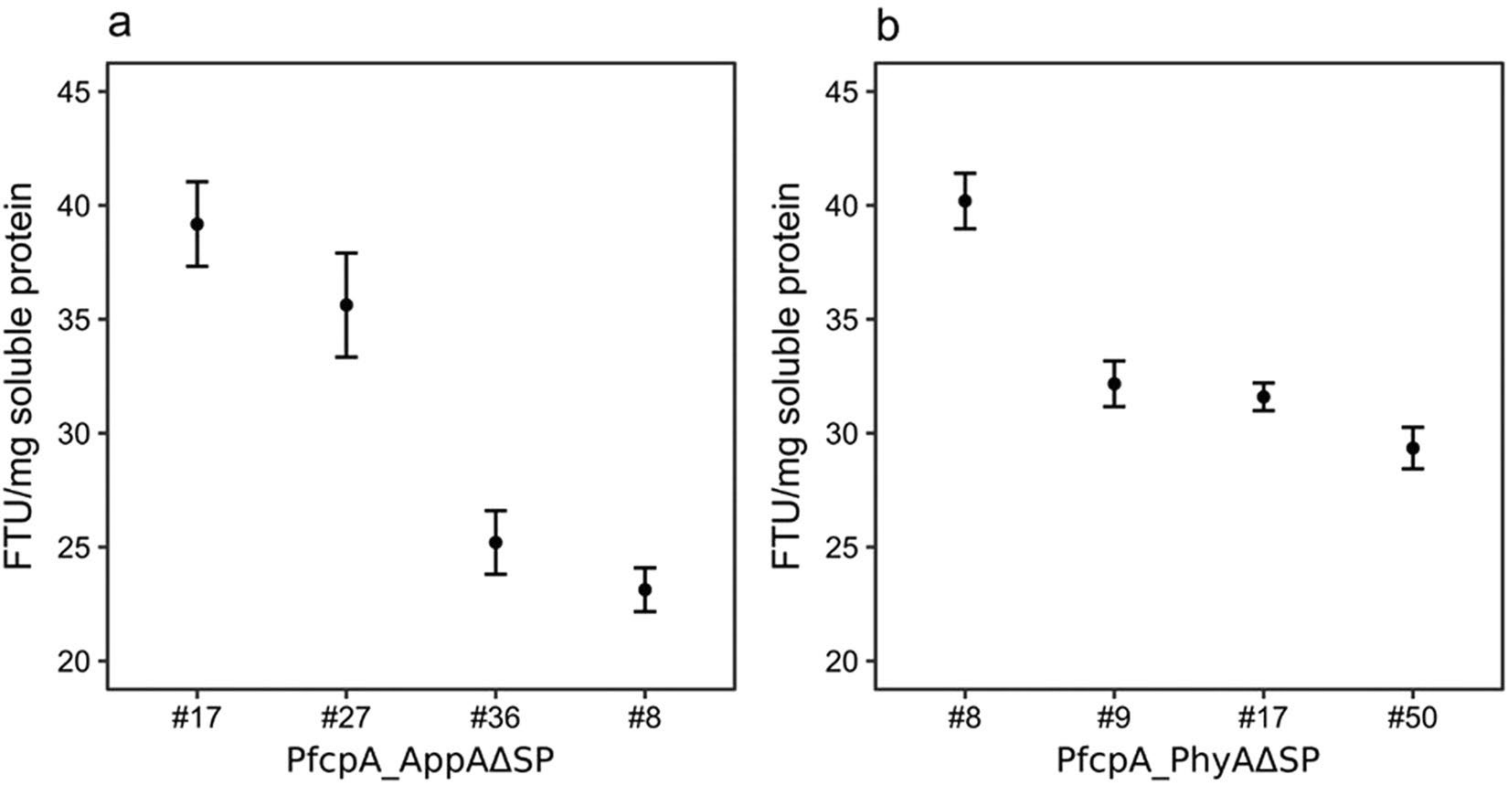
Phytase activities from transgenic *P*. *tricornutum* strains expressing mature peptides of *E*. *coli* AppA∆SP and *A*. *niger* PhyA∆SP. (**a**) AppA∆SP transformants. (**b**) PhyA∆SP transformants. Values are averages of measurements of three independent cultures, ±standard error. Mean assayable phytase activity in *P*. *tricornutum* WT lysates was previously established to be 3.13 (0.36 SEM) FTU/mg soluble protein (Fig. 3).

A 2.7-fold increase in group mean phytase activity was observed between strains of *P*. *tricornutum* expressing PfcpA_AppAΔSP (28.93 FTU/mg soluble protein; Fig. 4a) compared with the unmodified AppA group (10.86 FTU/mg soluble protein; Fig. 3a). Similarly, a three-fold increase was observed between PfcpA_PhyAΔSP (Fig. 4b) and PfcpA_PhyA (Fig. 3b) groups (33.33 and 10.87 FTU/mg soluble protein, respectively), demonstrating that removal of the putative N-terminal signal peptide resulted in the isolation of better phytase strains. Differences in mean phytase activity between the PfcpA_AppAΔSP and PfcpA_PhyAΔSP transformant groups were not statistically significant (p = 0.5981).

Individually, the best performing PfcpA_AppAΔSP and PfcpA_PhyAΔSP strains exhibited phytase activities of 39.18 and 40.2 FTU/mg soluble protein, respectively (Fig. 4), corresponding to a more than two-fold increase compared with their respective best unmodified PfcpA_AppA and PfcpA_PhyA phytase expressing strains (17.86 and 16.64 FTU/mg soluble protein; Fig. 3). Therefore, a successful strain engineering strategy for improved transgenic expression of phytase was validated, using the native fcpA promoter for expression of codon-optimized sequences corresponding to mature peptides AppA or PhyA. However, the isolation of best performing strains always required the screening of several transgenic lines and, therefore, remains time consuming.

### Fatty acid composition of phytase-expressing transgenic *P*. *tricornutum* strains accumulating enhanced levels of DHA

**Figure 5:**
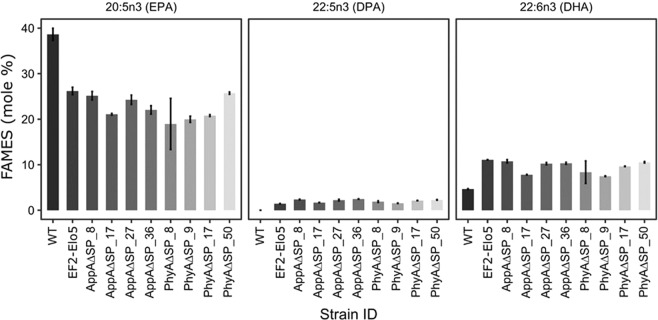
Fatty acid composition of WT, EF2_Elo5 and transgenic strains of *P*. *tricornutum* co-expressing the *O*. *tauri* ∆5 elongase and either AppA∆SP or PhyA∆SP under the fcpA promoter. Values are averages of measurements of at least three independent cultures, ±standard error.

Recently, we demonstrated that heterologous expression of *O*. *tauri* Δ5-elongase, OtElo5, in *P*. *tricornutum* resulted in enhanced levels of DHA accumulation in the transgenic strains^23^. In the present study, we co-expressed OtElo5 with either AppA or PhyA mature peptide sequences (Fig. 1) and analysed fatty acid compositions of the respective transgenic strains by gas chromatography coupled to flame ionisation detection (GC-FID) of fatty acid methyl esters (FAMEs). The total fatty acids (TFA) composition of OtElo5/phytase expressing strains was compared with WT and transgenic *P*. *tricornutum* expressing OtElo5 only (Supplementary Fig. S3). Consistent with previous results^23^, the main fatty acids in total lipid extracts of WT and transgenic cells were C16:1, C16:0 and EPA. Expression of OtElo5 under the EF2 promoter resulted in an increase in DHA content to an average of 11.09% (TFA). For groups of transgenic *P*. *tricornutum* strains co-expressing PfcpA_AppA∆SP or PfcpA_PhyA∆SP, the average DHA content was in the range 7.82% to 10.76% and 7.49% to 10.54% of total fatty acid (TFA) respectively (Fig. 5, Supplementary Table S1). Within the AppA∆SP group, the strain PfcpA_AppAΔSP_8 exhibited the highest DHA level (10.76% TFA) together with an average phytase activity of 23.13 FTU/mg soluble protein. Within the PfcpA_PhyA∆SP group, the highest DHA level was observed in PfcpA_PhyAΔSP_50 (10.54% TFA), with a corresponding phytase activity of 29.35 FTU/mg soluble protein. FAMEs analysis of the selected transgenic lines confirmed the presence of docosapentaenoic acid (DPA, 22:5n-3), the product of elongating activity of Δ5-elongase, in the range of 1.45–2.47% TFA; this fatty acid was not detected in WT cells. In transgenic strains, levels of EPA were decreased (18.97–25.74%) compared to 38.6% in the WT.

**Figure 6:**
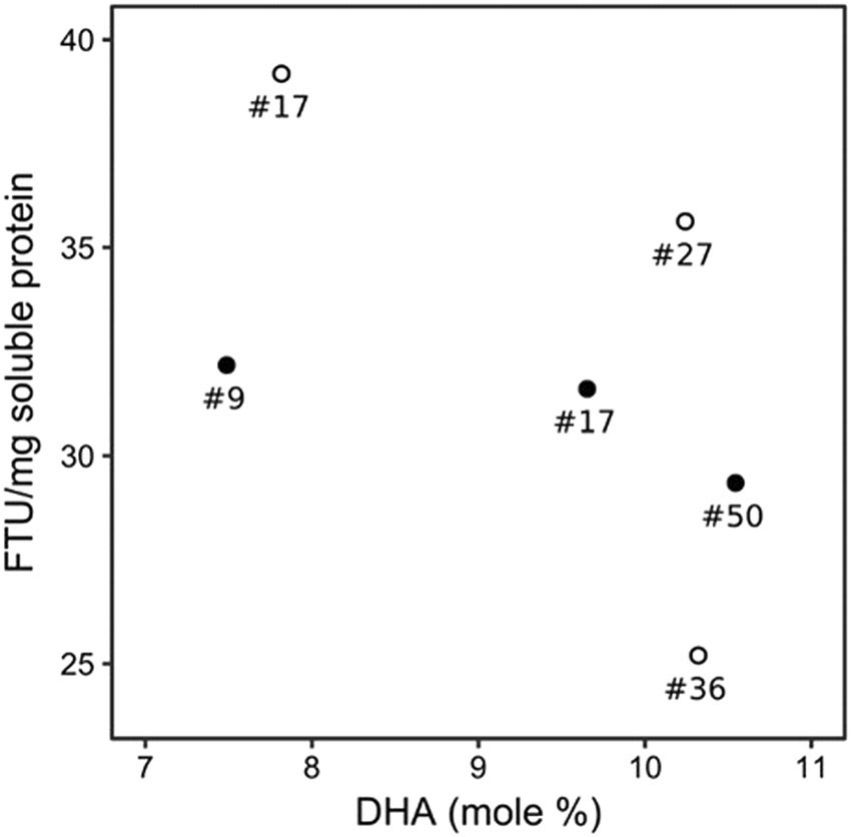
Comparison of phytase activity and DHA content of transgenic *P*. *tricornutum* strains. PfcpA-appA∆SP- 17, -27, -36 (open circles) and PfcpA-PhyA∆SP-9, -17, -50 (black circles).

Fatty acid profiles and phytase activities of key transgenic strains isolated during this work are summarized in Supplementary Table 1. Notably, there was no correlation between the top phytase expressers and the best DHA-accumulating strains. This observation further emphasizes the importance of screening multiple strains for the successful outcome of a multigene engineering in *P*. *tricornutum*. In fact, by plotting phytase activity against DHA content for each of the candidate strains (Fig. 6), it was possible to identify PfcpA_AppAΔSP_27 as the top performing strain when both metrics are considered (mean phytase activity 35.69 FTU/mg soluble protein and mean DHA content of 10.24% TFA). The total protein content remained unchanged among WT and the best transgenic strains (Supplementary Table S2).

Growth rates of transgenic lines were compared to those of the WT. Expression of the transgenes did not significantly alter cell growth rate in the different cell lines (Supplementary Fig. S4).

## Discussion

Over the last decade microalgae have attracted much attention for their potential to produce high value chemicals and recombinant proteins. The green alga, *Chlamydomonas reinhardtii*, became a focus of research on protein expression due to its easy cultivation and availability of molecular toolkit for genetic engineering^37^. Recently, it was reported that another microalgal system, the marine diatom *P*. *tricornutum*, can express recombinant proteins with high efficiency^21,22^.

In our previous studies, we demonstrated the efficacy of metabolic engineering of *P*. *tricornutum* to improve DHA content and large–scale cultivation of the resulting transgenic strain for industrial production of omega-3 LC-PUFA^23,24^. In this current work we have successfully engineered, for the first time, the marine diatom *P*. *tricornutum* to accumulate both high value omega-3 LC-PUFAs EPA and DHA and a recombinant protein, the enzyme phytase, to tackle the problem regarding phosphorus deficiency and general animal health. We have developed transgenic microalgal strains with enhanced levels of DHA expressing either bacterial AppA or fungal PhyA phytases. A crucial factor in the ongoing development of this technology was the observation that heterologous expression of transgenes did not impact cell growth rates.

To facilitate more accurate phytase activity measurements, we have optimized an assay method based on enzymatic hydrolysis of a phytic acid-lysozyme complex^33^. An accompanying prediction model for converting phytase activity from OD_600_ nm/min/mg to FTU was developed and used for the fast and accurate quantification of phytase activity in transgenic *P*. *tricornutum*.

We first tested transgenic expression of full-length codon-optimized versions of *E*. *coli* AppA and *A*. *niger* PhyA genes under different promoters. Previously, we have successfully used the light-inducible fcpA promoter for expression of OtElo5 gene in *P*. *tricornutum*^23^. However, there is a great demand for a constitutive gene expression system using a strong promoter to provide a high level of gene expression in transgenic *P*. *tricornutum* under any conditions. Therefore, the native EF2 promoter^29^ and viral CIP1 promoter^30^ were also tested. Comparison of phytase activities from *P*. *tricornutum* transformants expressing AppA and PhyA under the three different promoters (Fig. 3), indicated that promoter choice was influential on the level of phytase activity observed between each set of strains. *P*. *tricornutum* strains engineered with the native fcpA and CIP1 viral promoters showed the highest level of phytase’ activity. Interestingly, the expression of OtElo5 gene from the EF2 promoter resulted in the levels of EPA and DHA (Fig. 5) similar to that in transgenic strains where OtElo5 gene was under control of the fcpA promoter^23^. This may indicate that the OtElo5 only mediates the conversion of EPA to the DHA precursor, DPA, followed by highly efficient endogenous Δ4-desaturation of elongated product to generate DHA. By contrast, assayable phytase activity is always proportional to the quantity of protein expressed, and it is therefore a more accurate measure of promoter performance.

Improvements in phytase expression were further achieved through the deletion of the putative N-terminal signal peptide. Strain PfcpA_AppAΔSP_17 exhibited more than a two-fold increase in phytase activity (39.18 FTU/mg soluble protein), compared with the previous top *E*. *coli* AppA-expressing strain from the fcpA promoter group (17.86 FTU/mg soluble protein; Fig. 3a). There was a three-fold difference in group mean phytase activity between strains of *P*. *tricornutum* expressing the truncated PhyA (33.33 FTU/mg soluble protein), demonstrating that removal of the putative N-terminal signal peptide was beneficial for increasing phytase activity. This is in agreement with previous observations of Erpel *et al*.^16^, that only the use of mature peptide PhyA E288K without N-terminal secretion signal peptide, allowed trapping of the enzymatic activity within the intracellular space of the corresponding *C*. *reinhardtii* transgenic strain. Interestingly, when six microbial phytase genes were tested in the chloroplast of *C*. *reinhardtii*, only a full-length, codon-optimized version of the *E*. *coli* AppA gene was found to be functionally expressed^17^. These results suggest that the expression of microbial phytase genes in microalgae is regulated by additional factors such as protein targeting and retention, and that choice of algal platform organism might influence the required protein expression strategy^38^.

In the present study, we have demonstrated that we can produce high value omega-3 LC-PUFA and recombinant protein in the same strain of the marine diatom. *P*. *tricornutum*, thus contributing to the design of cost-efficient biorefinery process technology. The best engineered strain achieved up to 40,000 phytase activity units (FTU) per gram of soluble protein, which could treat up to 20 Kg of Atlantic Salmon feed^39^ and enhance levels of omega-3 LC-PUFA, further boosting the nutritional value of the diet with the addition of EPA and DHA. Further optimization of production strains and animal studies are underway to confirm the safety and assess the nutritional benefits of the generated transgenic strains and suitability for application within commercial feed applications.

## Methods

### Strains and growth conditions

*P*. *tricornutum* UTEX646 (Pt4) was grown in F/2 medium^40^ at 20 °C under white fluorescent lights’, constant illumination (60 µmol photons m^−2^ s^−1^) and agitated at 70 rpm. For the cell growth experiment samples were collected during the growth curve from day 0 to the late exponential phase (12–13 d). Cell concentration was measured by triplicate cell counts using a Cellometer Auto T4 Bright Field Cell Counter (Nexcelom bioscience).

### Plasmid design and cloning

A two-gene expression vector was constructed by modifying the previously described pPhOS2 vector, containing two multiple cloning sites (MCS)^23^. The codon optimized coding sequence for *O*. *tauri* OtElo5 was fused to the *P*. *tricornutum* fcpA terminator and inserted into position 1 in pPhOS2 vector under the control of the EF2 promoter^29^ generating pPt_EF2_Elo5 construct (Supplementary Fig. S1).

The coding sequences for the full- length phytase genes encoding AppA from *Escherichia coli* (GenBank accession number AAN28334) and PhyA from *Aspergillus niger* (GenBank accession number AAR08366) were used as templates to chemically synthesize (GenScript, Piscataway, NJ, USA) codon-optimized nucleotide sequences Pt_AppA and Pt_PhyA for expression in *P*. *tricornutum*. These codon-optimized sequences were flanked with *BamHI* and *XbaI* restriction sites and cloned into position 2 of pPt_EF2_Elo5 under control of either the native fcpA^31^ or EF2^29^ promoters, or the ClP1 viral promoter^30^ (Fig. 1).

In addition, mature protein sequences (without the N-terminal extracellular signal peptide, SP) for AppA and PhyA phytases were also created. Putative N-terminal signal peptides (SP) for both AppA and PhyA were identified with the SignalP web-server^41^. The predicted signal peptides comprise amino acids 1–27 and 1–33 of AppA and PhyA, respectively. Codon-optimized nucleotide sequences Pt_AppA and Pt_PhyA in constructs PfcpA_AppA and PfcpA_PhyA, respectively, were modified by deletion of a signal peptide by PCR using outward facing primer pairs EcMatF (5′-phos-ATGCAGTCCGAACCCGAACTCAA)/ MatR (5′-TTTGGTACCGGATCCGCG) for AppA and AnMatF (5′-phos-ATGCTCGCCGTCCCCGCCTCC)/MatR for PhyA. The forward primers EcMatF and AnMatF were designed to begin with the mature sequence immediately after the ATG, and with a 5′ phosphate group attached to facilitate self-ligation of the plasmid PCR product without a signal sequence. The deletions were confirmed by DNA sequencing.

### Biolistic transformation of *P*. *tricornutum*

*P*. *tricornutum* biolistic transformation was performed according to methods previously described^42^ using a Bio-Rad Biolistic PDS-1000/He Particle Delivery System (Bio-Rad Laboratories, Hercules, CA) with 1350 psi rupture discs. S550d gold carrier particles (Seashell Technology, California, USA) were coated with 5 µg of plasmid DNA for 3 individual microparticle bombardments, according to the manufacturer’s instructions. Bombarded cells were transferred onto 1.4% F/2 agar plates containing 75 µg/mL Zeocin (Thermo Fisher Scientific, Cat. No. R25005) and incubated at 20 °C. After 2–3 weeks, colonies were transferred to 1 mL of liquid F/2 medium containing 75 µg/mL Zeocin, for PCR screening of the transgenes.

### Lysis of *P*. *tricornutum* by sonication

Aliquots of 1 × 10^8^ algal cells were harvested by centrifugation at 3,000 rcf for 10 minutes at 20 °C. Pellets were resuspended in 500 µL of 1x SIGMAFAST^TM^ Protease Inhibitors, EDTA-free (Sigma Aldrich, Cat. No. S8830) in distilled water, transferred to 2 mL round-bottom microcentrifuge tubes and stored on ice. Cell suspensions were lysed by sonication at 7 microns amplitude, using a 3mm-wide tip, for 6x 10 second pulses separated by 30 seconds’ incubation on ice. Lysates were subsequently clarified by centrifugation using a bench-top centrifuge at 12,000 rcf for 10 minutes. Microscopic images of cell cultures after sonication were taken with a Zeiss Axiophot light microscope to confirm lysis of algal cells. Supernatants were separated by centrifugation (5 minutes, 16 000 *g*), transferred to sterile 2 mL round-bottom microtubes and stored on ice.

### Phytase turbidity assay

The phytase turbidity assay developed in this study for *P*. *tricornutum* cell extracts is based on a modified version of the protocol previously described by Tran *et al*.^43^, Assays were carried out in 96-well microtiter plate (MTP) format, in 120 µL final reaction volume consisting of 60 µL of substrate suspension (50 mM glycine pH 2.5, 0.4 mM phytic acid, 1.7 mg/mL lysozyme) and 60 µL of clarified cell lysate. Cell lysates were tested in serial dilutions. For positive control reactions, a water solution of a commercially-supplied phytase (EC: 3.1.3.26, Xi’an Rongsheng Biotechnology Co., Ltd. Shaanxi, China, 200 mg/mL) was used. To conduct the assay, 60 µL of cell lysate (or its dilution) was firstly add to the well, followed by 60 µL of substrate suspension. The final reaction was mixed thoroughly by pipetting and transferred to a microtiter plate reader pre-heated at 37 °C (FLUOstar Omega, BMG Labtech; Offenburg, Germany). The change in turbidity was monitored at 600 nm every 30 seconds for 20–60 minutes with shaking (double orbital) at 700 rpm for 5 seconds prior to each reading. Turbidity data was exported to R for analysis. Phytase activity (FTU/mg soluble protein) was calculated from the linear portion of each assay curve using a model of FTU regressed against change in OD_600_ nm. One phytase activity unit (FTU) is defined as the amount of enzyme which liberates 1 µmol inorganic phosphorus from an excess of substrate (here IP6-lysozyme) in 1 minute at 37 °C and pH 2.5. Total soluble protein content was determined by direct absorbance at 280 nm using a NanoDrop spectrophotometer (Thermo Fisher Scientific).

### Colorimetric determination of phosphate concentration

Data comprising the model for the prediction of P_i_ release was obtained using a modified ascorbic method for phosphate concentration^32,41^. The turbidity assay of commercial supplied phytase was stopped by adding 30 µL of 2.5 N HCl. 100 µL of the stopped reaction was added to 100 µL of 50 mM glycine-HCl pH 2.5 in a 2 mL microfuge tube, to which was added: 360 µL of 5% (w/v) SDS, 400 µL of 1.25% (w/v) ammonium molybdate in 2 M HCl and 40 µL of 1 g/L ascorbic acid to a final volume of 1 mL. The reaction was mixed briefly by vortex and incubated at room temperature for 30 minutes. Absorbance was read at 700 nm. A linear regression model to predict phosphate concentration was fitted to a plot of A_700_ nm for a series of phosphate standards in the 0.06 to 2.0 mM phosphate range. The adjusted R^2^ of the regression model was 0.9963 and the residual standard error was 0.01 mM phosphate on 11 degrees of freedom.

### Fatty acid analysis

10 mL aliquots of cells from the late exponential growth stage (4–5 × 10^7^ cells) were harvested by centrifugation at 4200 *g* for 10 minutes. Fatty acids were extracted and methylated as described previously^23^. Following methylation, the heptane fractions were concentrated and resuspended in heptane with 0.01% butylated hydroxytoluene. Methyl ester derivatives of the total fatty acids (FAMEs) were separated and quantified by gas chromatography flame ionisation detection (GC-FID) (Agilent 7890A) using an Agilent DB-23 column (30 m, 0.25 mm, 0.25 µm). Peaks were identified by comparison of retention times with Supleco 37 FAME Mix (Sigma) and methylated Qual Mix Menhaden fish oil (Larodan). 6.25 µg/mL of pentadecanoic acid (C15:0) and tricosanoic acid (C23:0) were used as internal standards.

### Statistical analysis

Statistical evaluation of phytase activity between groups of transgenic *P*. *tricornutum* expressing AppA or PhyA under three independent promoters (ClP1, EP2 and fcpA), plus a wild-type control, was tested using a factorial structure. However, the replication within the groups for the 3 by 2 factorial, after having accounted for the WT, was unequal and, therefore, precluded application of ANOVA, which requires a balanced experimental design. Thus, a linear mixed model was applied that accounted for the imbalance. The treatment terms were: (1) WT vs genetically-modified, (2) Between types having accounted for WT, (3) Between promoters having accounted for WT, (4) Interaction between types and promoters having accounted for WT and the main effects for the two factors. The model was fitted using the method of residual maximum likelihood as implemented in the GenStat (2015, 18th edition, VSN International Ltd, Hemel Hempstead, UK). Relevant means and standard error of the difference (SED) values for their comparison are based on the relevant degrees of freedom (df), thus allowing a least significant difference (LSD) value to be used for the comparisons of most biological interest. A natural log transformation was required to account for some heterogeneity of variance across the treatment combinations.

## Supplementary information

Dataset 1

## Data Availability

All data generated or analyzed during this study are included in this published article (and its Supplementary Information files).
