# Supplementary material for: Multifunctionalizing the marine diatom Phaeodactylum tricornutum for sustainable co-production of omega-3 long chain polyunsaturated fatty acids and recombinant phytase
Source: Sci Rep. 2019 Aug 7;9:11444. doi: 10.1038/s41598-019-47875-1 (PMC6686013; doi:10.1038/s41598-019-47875-1)
Supplement: Supplementary file 1 — Dataset 1 [file 41598_2019_47875_MOESM1_ESM.pdf]

Supplementary Data Information

Multifunctionilizing the marine diatom *Phaeodactylum tricornutum* for sustainable co-production of omega-3 long chain polyunsaturated fatty acids and recombinant phytase

Alex Pudney[1], Chiara Gandini[2], Chloe K. Economou[3], Richard Smith[2], Paul Goddard[4], Johnathan A. Napier[2], Andrew Spicer[1] and Olga Sayanova[2]*

*Author for correspondence - olga.sayanova@rothamsted.ac.uk

Supplementary Figure S1. **Plasmid map of the expression vector Pt_EF2_Elo5**.

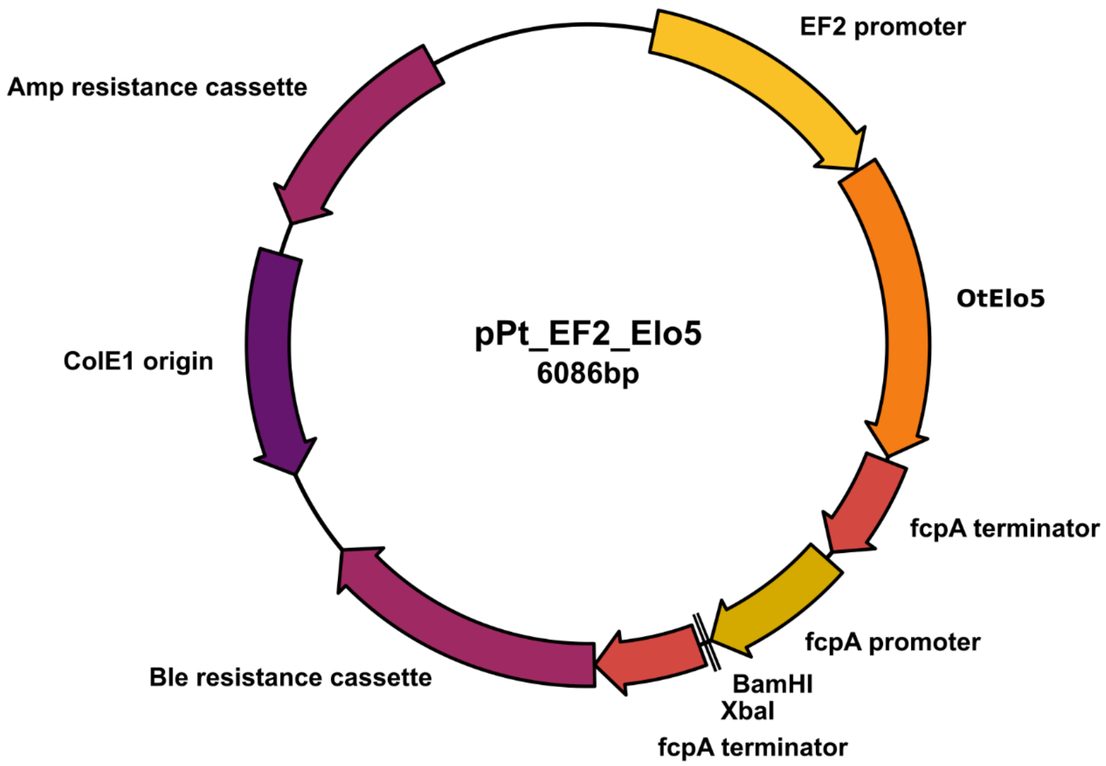

Supplementary Figure S2. Lysis of *P. tricornutum* cells by sonication. (**a**) *P. tricornutum* cells before sonication. (**b**) Visualization of cellular debris after sonication. Scale bar: 50µM.

a

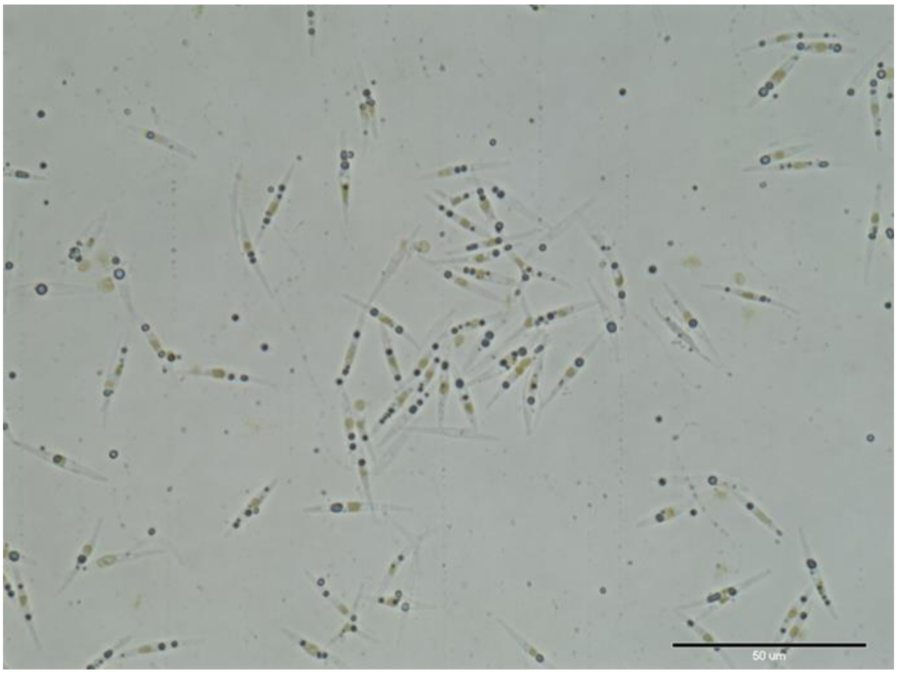

b

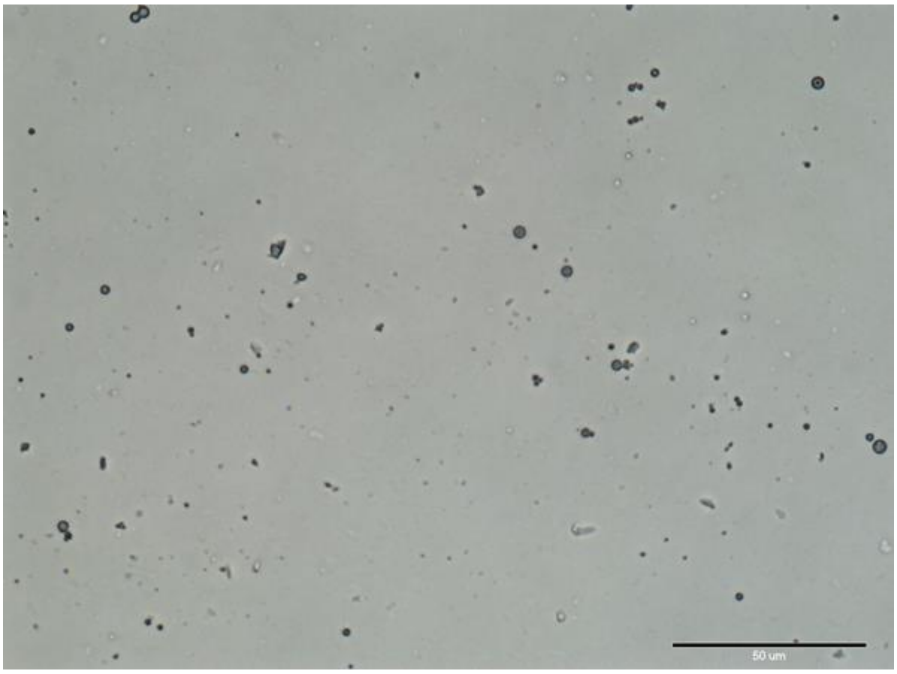

Supplementary Figure S3. Fatty acid composition of WT and transgenic strains of *P. tricornutum*.

Values are averages of measurements of at least three independent cultures, ± standard error.

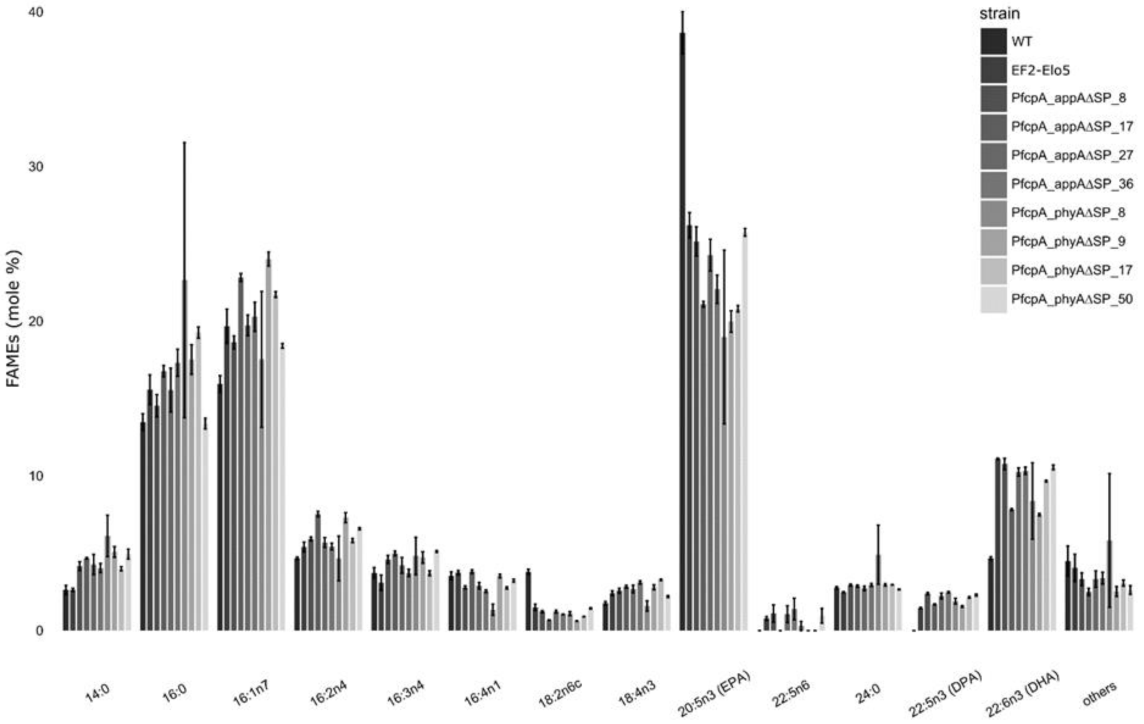

Supplementary Figure S4. Cellular growth of WT and transgenic *P. tricornutum* strains. Values are averages of measurements of three independent cultures, ± standard error.

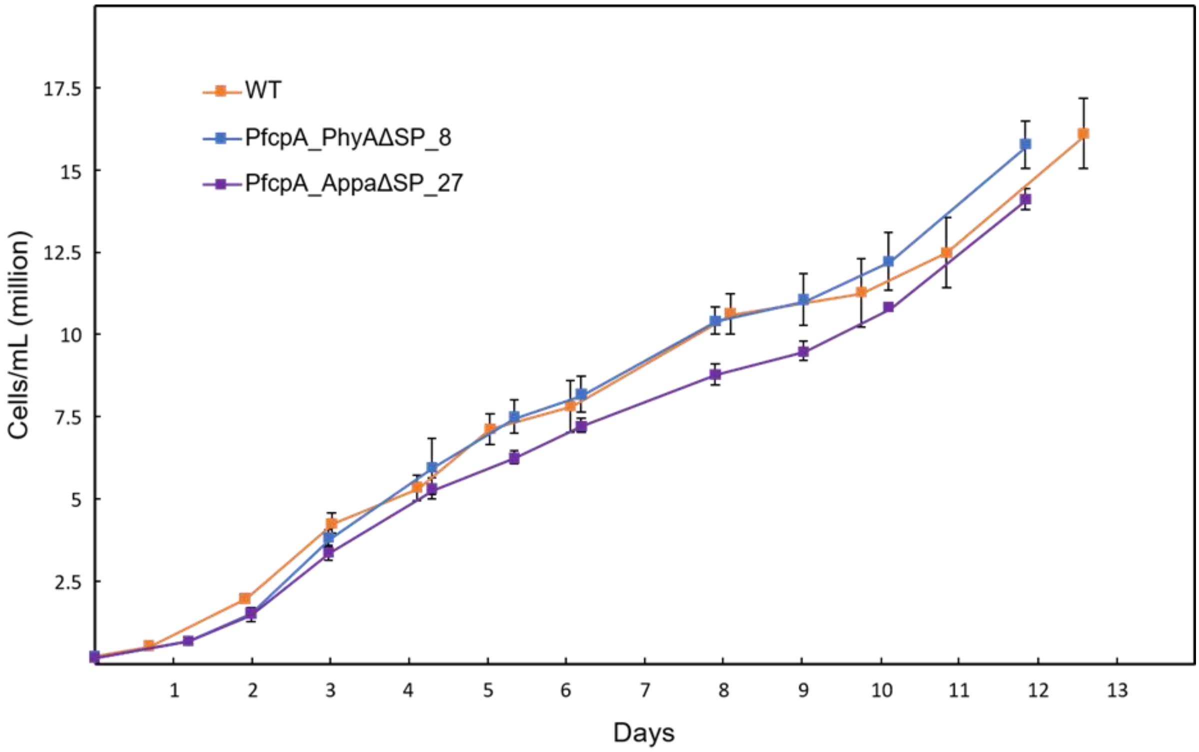

Supplementary Table 1. Fatty acid composition and phytase activities of WT and selected transgenic *P. tricornutum* strains. Values are averages of measurements of at least three independent cultures, ± standard error; ND: not detected; FTU/mg soluble protein.

Construct	FTU/mg	EPA (mole %)	DPA (mole %)	DHA (mole %)
WT	3.13 ±0.36	38.64 ±1.35	ND	4.67 ±0.10
Pt_EF2_Elo5	ND	26.2 ±0.82	1.45 ±0.04	11.09 ±0.04
PfcpA_appAΔSP_8	23.13 ±0.96	25.15 ±0.94	2.38 ±0.07	10.76 ±0.37
PfcpA_appAΔSP_17	39.18 ±1.86	21.09 ±0.19	1.69 ±0.03	7.82 ±0.06
PfcpA_appAΔSP_27	35.63 ±2.29	24.26 ±1.03	2.23 ±0.19	10.24 ±0.26
PfcpA_appAΔSP_36	25.2 ±1.40	22.05 ±0.91	2.47 ±0.05	10.32 ±0.23
PfcpA_phyAΔSP_8	40.2 ±1.22	18.97 ±5.62	1.9 ±0.19	8.37 ±2.47
PfcpA_phyAΔSP_9	32.17 ±1.00	19.98 ±0.69	1.54 ±0.06	7.49 ±0.08
PfcpA_phyAΔSP_17	31.59 ±0.61	20.8 ±0.20	2.14 ±0.05	9.65 ±0.05
PfcpA_phyAΔSP_50	29.35 ±0.91	25.74 ±0.25	2.29 ±0.06	10.54 ±0.15

Supplementary Table 2. The total soluble protein content of WT and transgenic PfcpA_appAΔSP_27 and PfcpA_phyAΔSP_9 strains. Values are averages of measurements of at least three independent cultures, ± standard error.

	Cells /mL	Protein (mg /mL)	Protein (µg /mL) /10^7 cells
WT	2.24 x 10^7	2.48 ± 0.068	110 ± 3
PfcpA_phyAΔSP_8	2.06 x 10^7	2.15 ± 0.102	104 ± 2
PfcpA_appAΔSP_27	2.28 x 10^7	2.33 ± 0.125	102 ± 4
